# Yeast Rpn4 Links the Proteasome and DNA Repair via *RAD52* Regulation

**DOI:** 10.3390/ijms21218097

**Published:** 2020-10-30

**Authors:** Daria S. Spasskaya, Nonna I. Nadolinskaia, Vera V. Tutyaeva, Yuriy P. Lysov, Vadim L. Karpov, Dmitry S. Karpov

**Affiliations:** 1 Center for Precision Genome Editing and Genetic Technologies for Biomedicine, Engelhardt Institute of Molecular Biology, Russian Academy of Sciences, Vavilov str. 32, 119991 Moscow, Russia; drspssk@gmail.com (D.S.S.); tutyaeva@gmail.com (V.V.T.); 2 Engelhardt Institute of Molecular Biology, Russian Academy of Sciences, 119991 Moscow, Russia; nioriss@gmail.com (N.I.N.); lysov@eimb.ru (Y.P.L.); karpov@eimb.ru (V.L.K.)

**Keywords:** 26S proteasome, Rpn4, DNA repair, *RAD52*, *RAD23*, *DDI1*, CRISPR/Cas9

## Abstract

Environmental and intracellular factors often damage DNA, but multiple DNA repair pathways maintain genome integrity. In yeast, the 26S proteasome and its transcriptional regulator and substrate Rpn4 are involved in DNA damage resistance. Paradoxically, while proteasome dysfunction may induce hyper-resistance to DNA-damaging agents, Rpn4 malfunction sensitizes yeasts to these agents. Previously, we proposed that proteasome inhibition causes Rpn4 stabilization followed by the upregulation of Rpn4-dependent DNA repair genes and pathways. Here, we aimed to elucidate the key Rpn4 targets responsible for DNA damage hyper-resistance in proteasome mutants. We impaired the Rpn4-mediated regulation of candidate genes using the CRISPR/Cas9 system and tested the sensitivity of mutant strains to 4-NQO, MMS and zeocin. We found that the separate or simultaneous deregulation of 19S or 20S proteasome subcomplexes induced *MAG1*, *DDI1*, *RAD23* and *RAD52* in an Rpn4-dependent manner. Deregulation of *RAD23*, *DDI1* and *RAD52* sensitized yeast to DNA damage. Genetic, epigenetic or dihydrocoumarin-mediated *RAD52* repression restored the sensitivity of the proteasome mutants to DNA damage. Our results suggest that the Rpn4-mediated overexpression of DNA repair genes, especially *RAD52*, defines the DNA damage hyper-resistant phenotype of proteasome mutants. The developed yeast model is useful for characterizing drugs that reverse the DNA damage hyper-resistance phenotypes of cancers.

## 1. Introduction

Genome stability is often threatened by the actions of various environmental and intracellular sources of DNA damage. Environmental DNA-damaging factors include sun radiation, Earth’s natural background radiation, industrial chemicals, and food mutagens [1]. The intracellular sources of DNA damage include mainly by-products of cellular metabolism (e.g., reactive oxygen or nitrogen species and alkylating agents). Each day, every cell experiences tens of thousands of DNA lesions of various types [2]. The most abundant are single-strand breaks (SSBs) and nitrogenous base losses, which account for approximately 75% of all detected DNA damage events resulting from dysfunctional DNA repair [2]. The direct actions of ionizing radiation, the replication of SSB-containing DNA fragments, and the effects of two SSBs near each other produce the most detrimental type of damage: DNA double-strand breaks (DSBs) [3]. If left unrepaired, some DNA lesions become mutations that largely contribute to the progression of pathologic states, such as neuro-degenerative diseases, cancer and ageing [4].

DNA damage induces a complex DNA damage response (DDR); as a part of this response, DNA repair pathways are activated [5]. These pathways restore genome integrity to prevent the accumulation of pathogenic mutations. The base excision repair (BER), nucleotide excision repair (NER), DNA mismatch repair (MMR), direct repair and translesion synthesis pathways address DNA damage on single strands. The homology-directed repair (HDR), non-homologous end joining (NHEJ) and microhomology-mediated end joining (MMEJ) pathways repair DSBs. A number of other cellular systems are activated as part of the DDR to facilitate and control the process of DNA repair. Control of the DNA repair enzyme levels is required to prevent the action of these enzymes upon the resolution of DNA damage, since the overexpression of some enzymes causes genome instability [6]. The ubiquitin-proteasome system is also activated during the DDR. Proteolysis may have several important functions during the DNA repair process. First, the 26S proteasome controls the levels of DNA repair proteins and enzymes. Its known substrates include Rad4 [7] and DNA polymerase η [8]. Second, proteolysis may serve as a driving force of the multistep DNA repair process by eliminating the components from preceding steps that may inhibit subsequent steps. Third, proteolysis is required to remove (1) proteins covalently attached to DNA [9] to give access to DNA damage sites, (2) stalled RNA-polymerase II [10] to reveal damaged DNA sites, and (3) histones to facilitate DNA dynamics [11]. Other components of the ubiquitin-proteasome system, e.g., ubiquitin and ubiquitin-like modifiers, such as SUMO, are also important players in DNA repair pathways. For example, the monoubiquitination of PCNA allows the loading of DNA translesion polymerases and the replication of damaged DNA strands [12]. Yeast Rpn4, a transcriptional master regulator and substrate of the proteasome [13,14], is another important player in the DDR and is involved in resistance to various DNA-damaging agents. Several lines of evidence support this. First, *RPN4* deletion sensitizes mutant yeast to DNA damage [15,16,17]. Second, the *RPN4* gene is induced, and the Rpn4 protein accumulates, upon the action of DNA-damaging agents [15,18,19]. Third, the promoters of a number of DNA repair genes have Rpn4 binding sites [19,20]. Fourth, the Rpn4-dependent induction of the BER pathway [20] or of NER and HDR [19] is observed upon DNA damage. These data suggest that both the proteasome and Rpn4 are positive regulators of DNA repair processes. Paradoxically, proteasome dysfunction, caused by inhibitors [19], mutations in proteasomal genes [21,22,23] or decreased expressions of essential proteasomal genes [19,24], leads to yeast hyper-resistance to DNA damage induced by camptothecin, 4-nitroquinoline-1-oxide (4-NQO) or methyl methanesulfonate (MMS). Although the deletion of the *RPN4* gene was also found to cause proteasome dysfunction, the mutant strains were sensitive to all DNA-damaging agents tested [15,16,17,25,26]. These data indicate that the proteasome may play a negative role in DNA repair, while Rpn4 is a positive regulator. Our previous data provide a clue to explain this paradox. We suggest that proteasome dysfunction stabilizes Rpn4 and that Rpn4-dependent DNA repair genes subsequently become overexpressed, after which the corresponding DNA repair pathways are hyper-activated. However, aside from the canonical Rpn4 binding site proteasome-associated control element (PACE), a number of DNA repair genes have PACE-like elements differing from PACE in one or two positions. It has been shown that Rpn4 has weak interactions with such elements [27,28]. These data question the direct involvement of Rpn4 in the regulation of DNA repair genes. Moreover, DNA repair genes should be regulated by several transcription factors; as such, the contribution of the Rpn4-mediated regulation of these genes to the DNA damage hyper-resistance phenotype of proteasome mutants is not clear.

Here, we show that the transcription of the key HDR gene *RAD52* is directly controlled by Rpn4. The Rpn4-dependent upregulation of DNA repair genes defines the DNA damage hyper-resistance phenotype of yeast with impaired proteasome function.

## 2. Results

### 2.1. Deregulation of Essential Proteasomal Genes Induces Hyper-Resistance to DNA Damage

**Figure 1:**
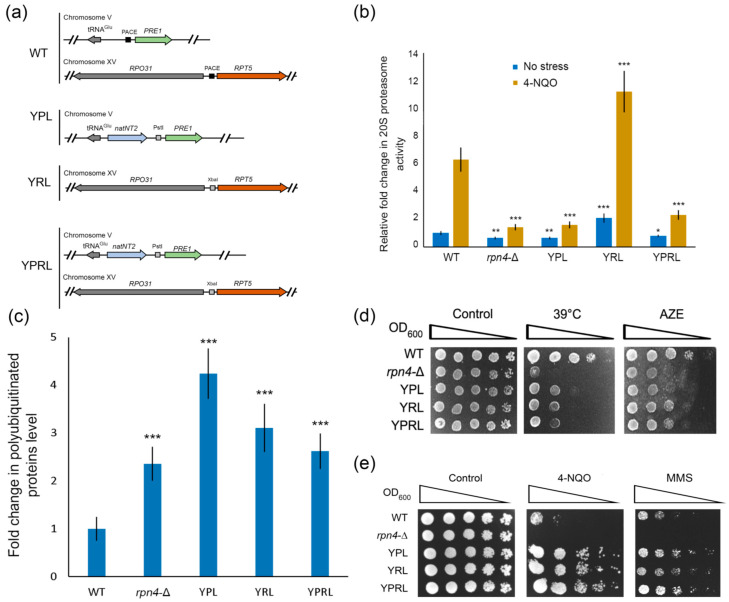
Yeast mutants with deregulated essential proteasomal subunits are hyper-resistant to DNA damage. (**a**) Schemes of the proteasomal mutant strains. (**b**) The 20S proteasome activity in proteasome-mutant strains. The 20S proteasome activity was measured in yeast exponential cultures under normal conditions or after 4-NQO treatment at a final concentration 1 µg/mL for 2 h. The relative signal for the wild-type (WT) strain was set to 1. The bar charts show the means (*n* = 3) ± SDs. Statistical significance: * *p* between 0.05 and 0.01, ** *p* between 0.01 and 0.005 and *** *p* < 0.001, according to Student’s *t* test. (**c**) Polyubiquitinated protein levels as quantified by ImageJ. The developed western blot picture is presented in Figure S1. The signal for polyubiquitinated proteins was normalized to the actin signal. The relative signal for the WT strain was set to 1. The values are the means (*n* = 3) ± SDs. *** *p* < 0.001, according to Student’s *t* test. (**d**) The proteasomal mutant strains were sensitive to proteotoxic conditions. The plates were incubated for 4 days under heat shock conditions or 3 days in the presence of 100 µg/mL L-azetidine-2-carboxylic acid (AZE). (**e**) The proteasomal mutant strains were hyper-resistant to DNA damage. The plates were incubated for 4 days at 30 °C. 4-NQO was used at a concentration of 0.75 µg/mL. MMS was used at a concentration of 0.017%.

We showed earlier that an impaired Rpn4-dependent regulation of genes encoding essential subunits of 20S proteolytic (*PRE1* in the YPL strain) [19] or 19S regulatory (*RPT5* or *RPT3* in the YRL or mRPT3 strain) [24] proteasome subcomplexes increases cell viability during acute and chronic exposure to DNA-damaging agents, such as MMS and 4-NQO. In this work, we obtained strains bearing PACE mutations in the promoters of both the *PRE1* and *RPT5* proteasomal genes to deregulate both 20S and 19S proteasome subcomplexes (YPRL strain, Figure 1a). The YPRL strain was obtained by exchanging the Rpn4 binding site PACE with an XbaI restriction site in the *RPT5* promoter on the genetic background of the YPL strain. Mutations were introduced by CRISPR/Cas9-induced template-dependent repair (see Materials and Methods). We confirmed that mutants with deregulated *PRE1* displayed decreased 20S proteasome activity (Figure 1b). In contrast, *RPT5* deregulation in the YRL strain led to increased 20S proteasome activity (Figure 1b). This result can be explained by the increased expression of 20S proteasome genes due to Rpn4 stabilization, as we previously showed [24]. Despite differences in 20S proteasome activity, all the mutant strains exhibited defects in 26S proteasome activity—they accumulated polyubiquitinated proteins upon heat shock (Figure 1c), and showed sensitivity to classic proteotoxic stresses such as heat shock and exposure to the toxic proline analogue L-azetidine-2-carboxylic acid, thus displaying impaired proteasome function (Figure 1d). However, the proteasome mutants were hyper-resistant to the DNA-damaging agents 4-NQO and MMS (Figure 1e). Notably, the *rpn4*-Δ mutant was sensitive to both proteotoxic and genotoxic agents. Therefore, while proteasome function was impaired in all strains, the strains with an intact *RPN4* gene showed hyper-resistance to DNA damage.

### 2.2. Identification of Rpn4-Dependent Genes Involved in the Cellular Response to DNA Damage

**Figure 2:**
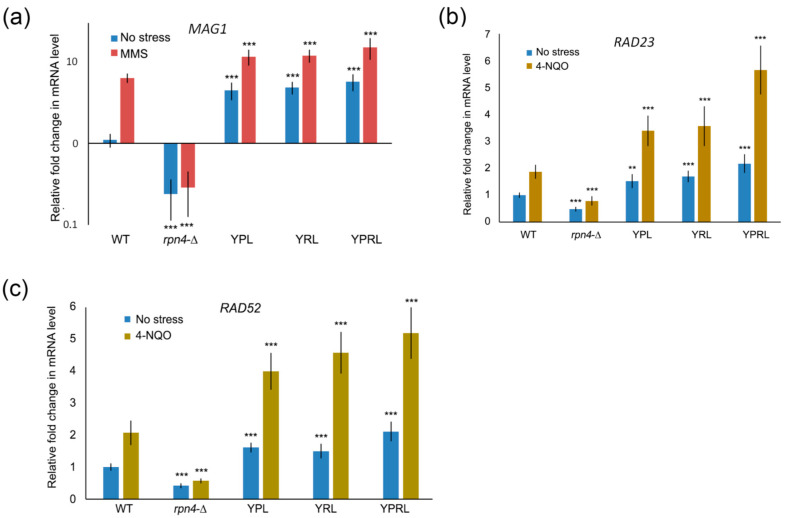
Rpn4-dependent DNA repair genes are upregulated in proteasome-mutant strains. The mRNA expression levels of *MAG1* (**a**), *RAD23* (**b**) and *RAD52* (**c**) were measured by RT-PCR under normal conditions, after 4-NQO treatment at a final concentration of 2 µg/mL for 45 min, or after MMS treatment at a final concentration of 0.2% for 30 min. *ACT1* was used as a reference. The relative mRNA level in the wild-type strain under normal conditions was set to 1. The bar charts show the means (*n* = 3) ± SDs. Statistical significance: ** *p* between 0.01 and 0.005 and *** *p* < 0.001, according to Student’s *t* test.

Rpn4 is a well-known proteasome substrate [13] that is stabilized upon deregulation of either the 19S or 20S proteasome subcomplexes [19,24]. We suggest that the upregulation of Rpn4-dependent DNA repair genes by stabilized Rpn4 contributes to the observed DNA damage hyper-resistance phenotype of proteasome mutants. To perform a systematic search for Rpn4-dependent DNA repair genes, we used the available data from several yeast genome-wide transcriptomic studies [29,30,31,32]. First, we created a list of genes that were differentially regulated (greater than or equal to twofold) in the wild-type strain upon exposure to at least one of the DNA-damaging agents, including ionizing radiation and compounds that methylate or oxidize DNA. Second, we created a list of 269 yeast genes with at least one of the following elements in their promoters that may serve as Rpn4 binding sites: PACE (5’-GGTGGCAAA-3’), MAG1-associated control element (MACE; 5’-GGTGGCGAA-3’) and RPN8-associated control element (RACE; 5’-AGTGGCAAA-3’) [33]. Dubious reading frames, retroelements, tRNAs and ARSs were excluded from the list (Table S4). The possibility that Rpn4 interacts with PACE-like elements was proposed previously [33], and was later shown experimentally [27,34]. We found that 207 out of the 269 genes were differentially expressed upon DNA damage (Table S4). Next, we selected genes that may function in DNA repair according to Saccharomyces Genome Database (SGD) annotation. We excluded from this list genes that we [19] and others [35] have previously shown to be regulated independently of Rpn4, despite having Rpn4 binding sites. We ultimately obtained the following list of 15 genes: *PRI1*, *EXO5*, *IRC20*, *SSL2*, *DEF1*, *BMH1*, *CUZ1*, *TMC1*, *HTB1*, *MSH3*, *ZWF1*, *MAG1*, *RAD52*, *RAD23* and *DDI*. We investigated whether some of these genes were expressed in an Rpn4-dependent manner. Real-time qPCR (RT-PCR) showed that *DEF1*, *MSH3* and *SSL2* were not dependent on Rpn4 (Figure S2). However, *MAG1*, *RAD23* and *RAD52* behaved as Rpn4-dependent genes. The expression of these genes was consistent with the stress resistance phenotypes of the mutant strains (Figure 1e); their expression was decreased in the *rpn4*-Δ strain under both normal and DNA stress conditions, and overexpressed in all proteasome mutants (Figure 2). These data indicate that the Rpn4-mediated overexpression of these genes may contribute to the observed phenotypes of the proteasome mutants.

### 2.3. Deregulation of Rpn4-Dependent DNA Repair Genes Sensitizes Mutant Yeast to DNA Damage

The identified Rpn4-dependent genes represent three different DNA repair pathways. *RAD23* encodes a protein that functions in NER [36,37]. The *RAD23* promoter contains the PACE-like element MACE, and Rpn4 is involved in the regulation of this gene [18,20]. *MAG1* encodes 3-methyl-adenine DNA glycosylase, which initiates BER. The *MAG1-DDI1* bidirectional promoter contains two MACEs. Although the interaction of Rpn4 with MACE in the *MAG1-DDI1* promoter has not been shown by classical methods [38], Rpn4 is required for stress-induced *MAG1* expression [20,39]. Earlier, using a highly sensitive DNA adenine methyltransferase identification (DamID) assay, we showed that Rpn4 interacts with the *MAG1-DDI1* promoter, and we used a lacZ assay to show that the proximal MACE largely contributes to Rpn4-dependent *MAG1* regulation [39]. *RAD52* is a key member of the group of *RAD52* epistasis genes, and is involved in DSB repair and recombination in yeast [40]. The *RAD52* promoter contains the PACE-like element RACE at the −72 nt position relative to the start codon. Therefore, this site is located within the typical region for PACE or PACE-like elements in proteasomal genes and other Rpn4-dependent genes [41,42]. Earlier, we showed that *RAD52* expression is regulated in an Rpn4-dependent manner [19].

**Figure 3:**
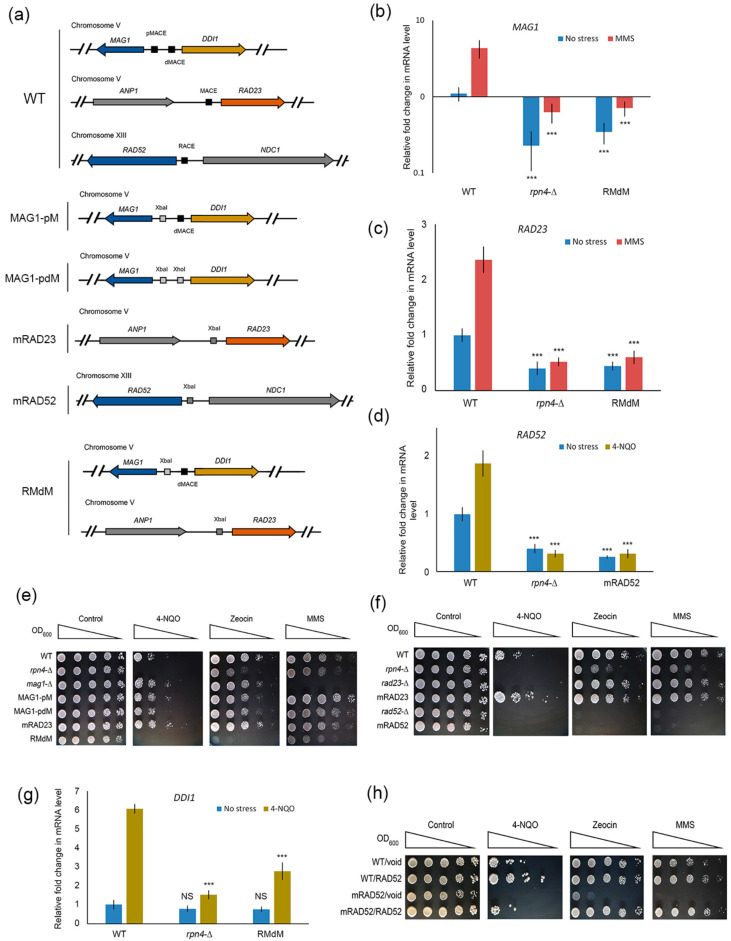
Deregulation of Rpn4-dependent DNA repair genes sensitizes yeast to DNA damage. (**a**) Scheme of yeast mutants bearing Rpn4 binding site (MACE or RACE) mutations in the promoter regions of the *MAG1-DDI1*, *RAD23* and *RAD52* DNA repair genes. Mutations were introduced into the yeast genome using CRISPR/Cas9-induced template-dependent repair. Both normal and stress-induced expression of *MAG1* (**b**), *RAD23* (**c**) and *RAD52* (**d**) were impaired in strains with mutated Rpn4 binding sites. Exponentially grown cultures were treated with 2 µg/mL 4-NQO for 45 min or 0.2% MMS for 30 min at 30 °C. mRNA levels were measured by RT-PCR and normalized to *ACT1*. The relative mRNA level in the wild-type (WT) strain under normal conditions was set to 1. The bar charts show the means (*n* = 3) ± SDs. Statistical significance: *** *p* < 0.001, according to Student’s *t* test; (**e**–**f**) Results of the stress resistance test for mutant strains with deregulated *MAG1*, *RAD23* and *RAD52* genes. DNA-damaging agents were used at the following concentrations: 4-NQO, 0.75 µg/mL; MMS, 0.01%; and zeocin, 250 µg/mL. The plates were incubated for 4 days at 30 °C; (**g**) RT-PCR showed that the mutation of MACE proximal to the *MAG1* gene in the *MAG1-DDI1* bidirectional promoter decreased the expression of *DDI1* under stress conditions. The relative mRNA level in the WT strain under normal conditions was set to 1. The bar charts show the means (*n* = 3) ± SDs. Statistical significance: NS, non-significant; *** *p* < 0.001, according to Student’s *t* test. (**h**) A plasmid with *RAD52* under the control of the native promoter restored resistance to DNA damage in the mRAD52 mutant strain. DNA-damaging agents were used at the following concentrations: 4-NQO, 0.75 µg/mL; MMS, 0.012%; and zeocin, 250 µg/mL. The plates were incubated for 5 days at 30 °C.

The promoters of these DNA repair genes contain binding sites for other transcription factors. Therefore, the contribution of Rpn4 to the regulation of these genes and the DNA damage resistance phenotype is unclear. To elucidate the role of Rpn4, we created mutant yeast strains bearing mutations of the PACE-like elements in the promoters of genes of interest (Figure 3a). The GC-rich part of these elements was replaced with restriction endonuclease sites by CRISPR/Cas9-induced template-dependent repair. Since the bidirectional *MAG1-DDI1* promoter has two MACE sites, we obtained mutant strains with mutations of MACE proximal to *MAG1* (MAG1-pM) and both proximal and distal MACE (MAG1-pdM). Additionally, we obtained a double mutant strain, RMdM, bearing mutations of proximal MACE in the *MAG1-DDI1* promoter and MACE in the *RAD23* promoter. RT-PCR showed that in the mutant strains, both the normal and DNA damage-induced expression of *RAD52* and *RAD23* returned to the levels observed in the *rpn4*-Δ strain (Figure 3b,c). While MACE-mutant *MAG1* expression under normal conditions was several-fold higher than that in the *rpn4*-Δ strain, the stress-induced level of *MAG1* in the MACE-mutant was indistinguishable from that in the *rpn4*-Δ strain (Figure 3d). Thus, we conclude that the DNA damage-mediated induction of *RAD52*, *RAD23* and *MAG1* DNA repair genes is largely dependent on Rpn4, which acts via PACE-like elements. Next, to elucidate the contribution of the Rpn4-dependent regulation of DNA repair genes to the sensitivity of the mutant strains, we tested their resistance to DNA-damaging agents. In addition to 4-NQO, which produces NER substrates, and MMS, which creates BER substrates, we used the antibiotic zeocin, which induces the formation of DSBs and activates several HDR genes, including *RAD52*, which is absolutely required for zeocin stress survival [43]. According to the results obtained, *RAD52* deletion or deregulation renders mutant cells sensitive not only to zeocin but also to 4-NQO or MMS. Earlier, it was shown that *RAD52* is also important for yeast resistance to another DNA alkylating agent, MNNG [44], and to oxidative DNA damage [45]. Active derivatives of metabolized 4-NQO may generate reactive oxygen species that, in turn, induce oxidative DNA damage [46]. Moreover, closely opposed SSBs, as intermediates in NER or BER pathways, may produce highly toxic DSBs [3], which are substrates for Rad52 and other components of HDR. While *MAG1* deletion renders cells hypersensitive to MMS, *MAG1* deregulation has no effect on yeast’s sensitivity to MMS (Figure 3e). Compared with the *rad23-∆* mutant, the mutant strain mRAD23 with deregulated *RAD23* (Figure 3e) was also resistant to 4-NQO (Figure 3f). A possible explanation for such phenotypes among *MAG1* and *RAD23* mutants is that despite decreased expression, the quantity of the produced enzyme is sufficient to cope with the corresponding DNA damage. Unexpectedly, the double mutant RMdM was sensitive to all three agents (Figure 3e). Since *MAG1* is not required for 4-NQO resistance (Figure 3e), we suspect that its neighboring gene *DDI1* contributes to the RMdM phenotype. Indeed, RT-PCR showed that *DDI1* was an Rpn4-dependent gene, and its expression was decreased in the RMdM mutant upon 4-NQO stress (Figure 3g). *DDI1* encodes a multi-domain protein with a ubiquitin-like domain, a ubiquitin-interacting domain and an aspartic protease domain [47]. *DDI1* has several molecular functions, including participation in the DDR response to MMS [48,49]. Interestingly, the mRAD52 mutant displayed the most severe phenotype of sensitivity to DNA damage, almost identical to the phenotype of the *rad52*-∆ strain (Figure 3f). To prove that the mRAD52 phenotype was caused by *RAD52* deregulation rather than by some CRISPR/Cas9 off-target effect or spontaneous mutations, we transformed mRAD52 with a plasmid encoding *RAD52* under the control of its native promoter. Indeed, *RAD52* complementation restored mRAD52’s resistance to DNA damage (Figure 3h). These data suggest that Rpn4 is a critical regulator of *RAD52* function. Notably, an extra copy of *RAD52* led to the hyper-resistance of the wild-type strain to 4-NQO (Figure 3h), which suggests that the overexpression of DNA repair genes may provide hyper-resistance to DNA-damaging agents.

Additionally, we measured the 20S proteasome activity, and found that it is not altered in the mutants with impaired Rad52 function (Figure S3). These results suggest that the severe sensitivity to DNA damage of *RAD52* mutants is independent of proteasome proteolytic function.

### 2.4. Rpn4 Directly Regulates RAD52 via a PACE Variant

**Figure 4:**
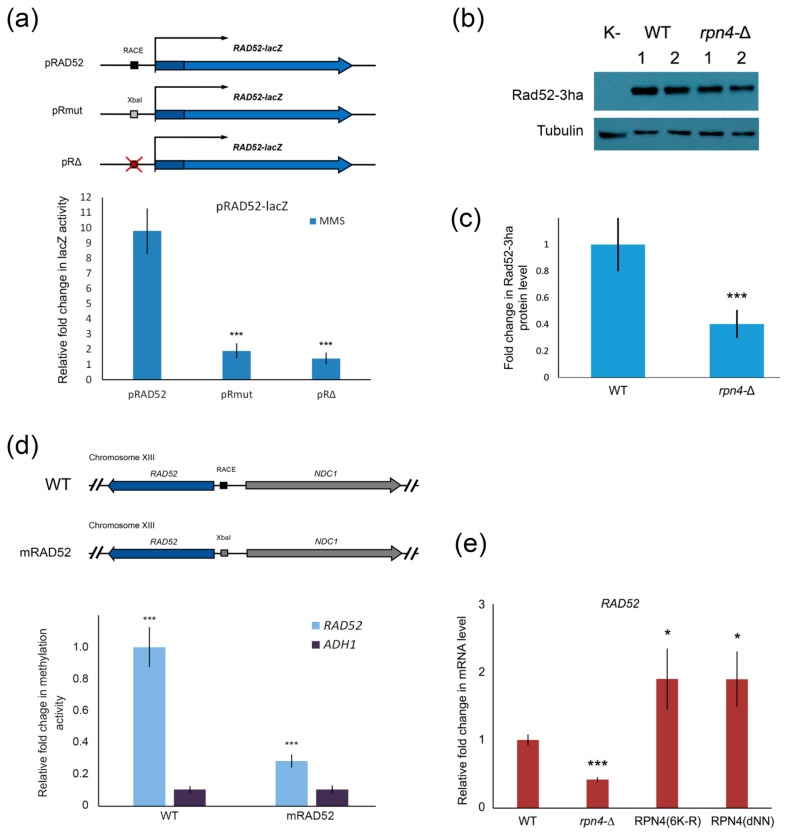
Rpn4 regulates *RAD52* directly via RACE. (**a**) RAD52-lacZ translational fusion reporters showed that the RACE element is required for DNA damage-mediated induction of *RAD52*. Schemes of the RAD52-lacZ reporters used are given. pRAD52 bears the *RAD52* promoter and 5′ part of the *RAD52* ORF fused in-frame to the *lacZ* gene. pRmut differs from pRAD52 only by substitution of the 5′-AGTGGC-3′ part of the RACE element with the XbaI site (5′-TCTAGA-3′). The pRΔ construct differs from pRAD52 by deletion of the RACE element. The lacZ activity is reported relative to that of the pRΔ construct. The bar charts show the means (*n* = 3) ± SDs. Statistical significance: NS, non-significant; *** *p* < 0.001, according to Student’s *t* test. (**b**) Western blot analysis of Rad52-3ha levels in the wild-type (WT) and *rpn4*-Δ strains. Numbers designate independent colonies. The full image of the developed western blot is presented in Figure S4. (**c**) Rad52-3ha levels quantified by ImageJ software. The signal for Rad52-3ha was normalized to the tubulin signal. The relative signal for the WT strain was set to 1. The values indicate the means (*n* = 4) ± SDs. *** *p* < 0.001, according to Student’s *t* test. (**d**) RACE mutation inhibited the Rpn4 interaction with the *RAD52* promoter. The methylation signal of the Dam-Rpn4 chimeric reporter protein was normalized to the signal from the mutant reporter Dam-Rpn4(C-A) with impaired Rpn4-binding activity. The *ADH1* gene is not an Rpn4 target and was used as a negative control. The relative Dam-Rpn4 signal on the *RAD52* promoter in the WT strain was set to 1. The bars and error bars are the means (*n* = 3) ± SDs. *** *p* < 0.001, according to Student’s *t* test. (**e**) Rpn4 stabilization was sufficient to induce *RAD52*. The mRNA level of *RAD52* in the WT strain expressing stabilized Rpn4 forms was measured using RT-PCR. Rpn4 was stabilized by the deletion of degradation signals (RPN4-dNN) or the mutation of all six N-terminal lysines that contribute to Rpn4 polyubiquitination (Rpn4(6K-R)). The *RAD52* mRNA level in the WT strain was set to 1. The values are the means (*n* = 3) ± SDs. * 0.01 < *p* < 0.05, *** *p* < 0.001, according to Student’s *t* test.

Little is known about the transcriptional regulation of *RAD52*, so we sought to clarify whether Rpn4 regulates this gene directly or indirectly. Using a lacZ reporter system, we found that RACE deletion or substitution with the XbaI restriction site impaired MMS-induced Rad52 activation (Figure 4a). Additionally, we used western blotting to show that the Rad52 level is decreased in the *rpn4*-Δ strain (Figure 4b,c). To investigate the ability of Rpn4 to bind the RACE sequence in the *RAD52* promoter, we performed DamID. According to the DamID results, Rpn4 is recruited to the wild-type *RAD52* promoter (Figure 4d). To confirm that Rpn4 binds to the *RAD52* promoter via the RACE sequence, we created a mutant strain with a RACE-to-XbaI mutation using the CRISPR/Cas9 system. Indeed, Rpn4 was not recruited to the mutated *RAD52* promoter (Figure 4d). Thus, we conclude that Rpn4 regulates *RAD52* directly via interaction with RACE. Rad52 expression at the protein level is well correlated with its mRNA level. To test whether Rpn4 stabilization is sufficient for *RAD52* induction, we measured the *RAD52* mRNA levels in the wild-type strain expressing Rpn4-stabilized forms (Figure 4e). The RT-PCR results proved that Rpn4 stabilization is sufficient for *RAD52* overexpression. To our knowledge, Rpn4 is the first described transcriptional regulator for yeast *RAD52*.

### 2.5. Impaired Rpn4-Dependent Regulation of DNA Repair Genes Restores the Sensitivity of Proteasome Mutants to DNA Damage

**Figure 5:**
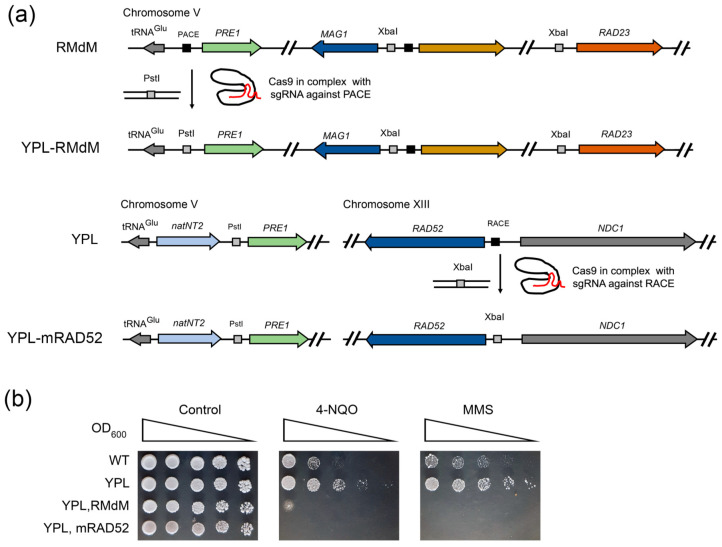
Deregulation of Rpn4-dependent DNA repair genes causes sensitivity to DNA damage in proteasome-mutant strains. (**a**) Schemes for the creation of mutants with deregulation of both *PRE1* and DNA repair genes. Mutations were introduced by CRISPR/Cas9-induced template-dependent repair. (**b**) The deregulation of DNA repair genes on a YPL background sensitizes mutant yeast to DNA damage. DNA-damaging agents were used at the following concentrations: 4-NQO, 0.7 µg/mL; MMS, 0.016%. The plates were incubated for 4 days at 30 °C.

Our results suggest that the Rpn4-dependent induction of *RAD23* together with *DDI1* or *RAD52* alone is crucial for yeast resistance to DNA damage. To test whether the Rpn4-dependent overexpression of DNA repair genes provides proteasome mutants with hyper-resistance to DNA damage, we created two mutant strains using the CRISPR/Cas9 system (Figure 5a). In the first strain, YPL-RMdM, we introduced a PACE mutation in the *PRE1* promoter on the genetic background of the RMdM strain. In the second strain, YPL-mRAD52, we introduced a RACE mutation in the *RAD52* promoter on the genetic background of the YPL strain. The presence of mutations was verified by PCR amplification of the mutated promoters, followed by restriction analysis (Figure S5). Deregulation of both *PRE1* and *RAD52* genes in the yeast double mutants was confirmed by RT-PCR (Figure S6). The stress resistance test showed that the obtained mutants were sensitive to DNA damage, with the YPL-mRAD52 strain displaying the most severe phenotype (Figure 5b). Our results suggest that the Rpn4-dependent overexpression of DNA repair genes, especially *RAD52*, defines the DNA damage hyper-resistance phenotype of proteasome mutants.

### 2.6. CRISPR-Mediated RAD52 Repression Decreases the Resistance of Proteasome Mutants to DNA Damage

**Figure 6:**
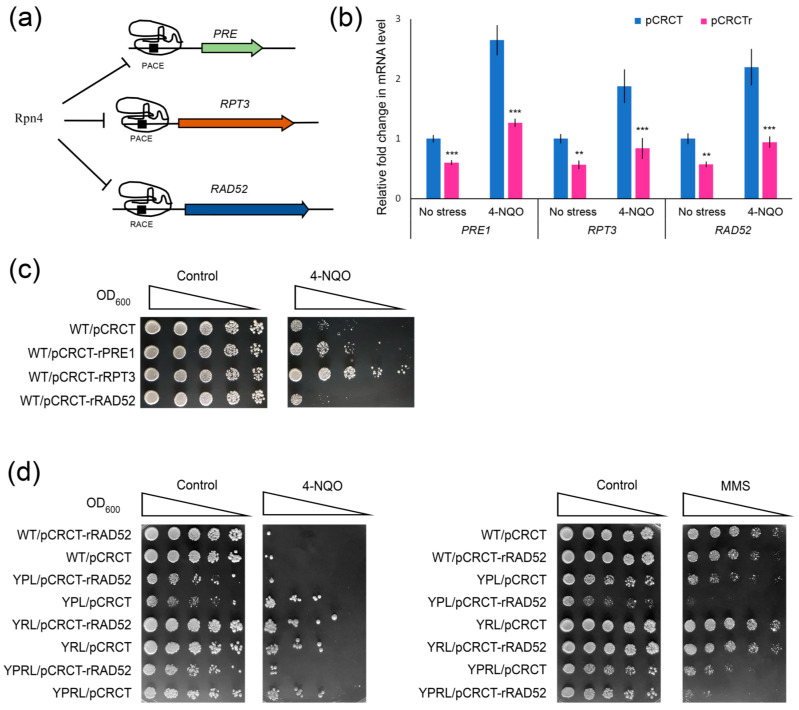
CRISPR/Cas9-mediated repression of proteasomal and *RAD52* genes. (**a**) Scheme of the experiment. In complex with a short sgRNA, SpyCas9 binds to the PACE or PACE-like element, thereby inhibiting Rpn4 binding. (**b**) RT-PCR confirmed the CRISPR/Cas9-mediated repression of proteasomal and *RAD52* genes in the wild-type (WT) strain both under normal conditions and upon 4-NQO treatment (2 µg/mL for 45 min). The mRNA level of the corresponding gene in the WT strain transformed with the empty pCRCT vector was set to 1. pCRCTr denotes the WT strain transformed with the pCRCT plasmid bearing a short spacer against PACEs in the *PRE1* or *RPT3* proteasomal genes or *RACE* in the RAD52 promoter. The values are the means (n = 3) ± SDs. ** 0.05 < *p* < 0.001, *** *p* < 0.001, according to Student’s *t* test. (**c**) CRISPR/Cas9-mediated repression of the proteasomal gene *PRE1* or *RPT3* induced hyper-resistance to 4-NQO, while *RAD52* repression sensitized yeast to 4-NQO. (**d**) CRISPR/Cas9-mediated repression of *RAD52* sensitized yeast mutants with deregulated proteasomal subunits to DNA-damaging agents. Concentrations of the DNA-damaging agents used: 4-NQO, 0.85 µg/mL; MMS, 0.015%. The plates were incubated for 5 days at 30 °C.

Mutant yeast may adapt to mutations in important genes. To confirm the results of the mutation experiments through an orthogonal approach, we performed a CRISPR repression experiment. Taking advantage of naturally occurring protospacer-adjacent motifs (PAMs) in the Rpn4 binding sites, we designed short (14 nt) spacers to target PACEs or RACEs in the promoters of *PRE1*, *RPT3* or *RAD52* (Figure 6a). SpyCas9 in complex with a short spacer should bind the target element, but not cleave it. Therefore, SpyCas9 should interfere with the Rpn4-dependent regulation of the corresponding genes. Recently, we used such an approach to inhibit the Rpn4-dependent expression of *PRB1*, one of the key autophagy genes [50]. RT-PCR confirmed the repression of the CRISPR-targeted genes (Figure 6b). The CRISPR-mediated repression of proteasomal genes in the wild-type strain clearly reproduced the 4-NQO hyper-resistant phenotype of proteasome mutants (Figure 6c). In addition, *RAD52* repression in the wild-type strain (Figure 6c) as well as in the proteasome mutants (Figure 6d) decreased yeast resistance to DNA damage. Notably, *RAD52* repression in the YRL strain had mild effects on YRL resistance to DNA damage. Since the 19S regulatory subcomplex may participate in the DNA repair process independently of the 20S proteasome [51,52], this could complicate the mutant yeast response to some types of DNA damage.

### 2.7. Dihydrocoumarin (DHC) Reverses the DNA Damage Hyper-Resistance Phenotype of Proteasome Mutants

**Figure 7:**
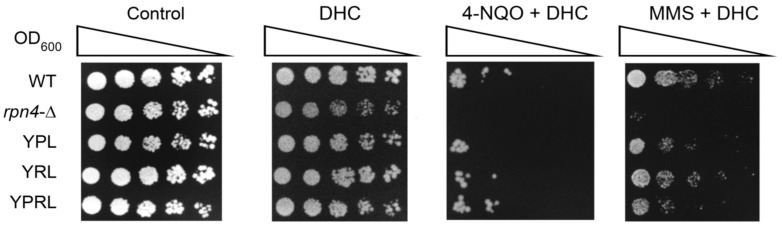
DHC reverses the DNA damage hyper-resistance phenotype of proteasome mutants. Concentrations of chemicals used: DHC, 3 mM; 4-NQO, 0.45 µg/mL; MMS, 0.0175%. The plates were incubated for 4 days at 30 °C.

DHC, a natural compound found in *Melilotus officinalis* (sweet clover), is characterized as an inhibitor of the yeast NAD-dependent histone deacetylase (HDAC) Sir2 [53]. Recently, it has been shown that DHC, through HDAC inhibition, suppresses yeast HDR via *RAD52* repression [54]. We sought to test whether the DHC-mediated chemical inhibition of *RAD52* expression in proteasome mutants sensitizes them to DNA damage. Indeed, the stress resistance test showed that in the presence of DHC, the proteasome mutants formed colonies with, at best, the same rate as the wild-type strain, thereby showing no DNA damage hyper-resistance phenotype (Figure 7).

## 3. Discussion

We found that the Rpn4-dependent overexpression of the DNA repair genes *RAD23*, *DDI1,* and especially *RAD52* provides hyper-resistance to various DNA-damaging agents in yeast mutants with impaired 26S proteasome functions. The genetic, epigenetic or chemical inhibition of *RAD52* expression restores sensitivity to DNA damage in the proteasome-mutant strains.

The characterization of the cellular response to DNA damage, particularly the mechanisms of DNA repair, has important medical applications for understanding and treating pathologies such as cancer, neurodegenerative diseases and ageing [55]. Moreover, it is important to improve the genome editing technologies that rely on cellular DNA repair pathways [56].

We here show that the Rpn4-dependent regulation of *RAD23* and *DDI1* or of *RAD52* alone is crucial for yeast survival upon exposure to various DNA-damaging agents. Both *RAD23* and *DDI1* encode highly conserved proteins that possess ubiquitin-associated (UBA) and ubiquitin-like (UBL) domains. Therefore, they function as adaptor proteins in the ubiquitin-dependent protein degradation pathways by delivering substrates to the proteasome [57,58]. However, these proteins also have distinct functions in DNA repair pathways. Rad23 in complex with Rad4 senses DNA damage [36]. Ddi1 may function as an aspartic protease and assist in the removal of proteins cross-linked to DNA, e.g., the topoisomerase I cleavage complex (topoisomerase I-DNA covalent complex, TopIcc) and RNA polymerase II [48]. Additionally, Rad23p and Ddi1p may heterodimerize via their UBA domains [59], which may indicate that they exert coordinated actions. We found that the deregulation of *RAD23* or *DDI1* alone resulted in no visible phenotype (Figure 3e,f). However, the deregulation of both genes sensitized yeast to all DNA-damaging agents tested, especially 4-NQO (Figure 3e). Since both proteins may function as proteasome adaptors, they may have degenerated functions, e.g., the degradation of proteins that become ubiquitinated proteasome substrates upon DNA damage, and thus each may compensate for a decrease in the concentration of the other. It is tempting to speculate that these proteins may function in the same NER pathway. NER may participate in the removal of DNA–protein crosslinks [60]. However, NER cannot operate on peptides or proteins larger than 10 kDa [61]. Thus, TopIcc or stalled RNA polymerase II cannot be processed by NER directly. Ddi1-induced proteolytic cleavage of TopIcc or RNA polymerase II may remove the obstacles and permit the Rad23/Rad4 complex to sense the damaged DNA locus and initiate NER. 4-NQO may stimulate the trapping of Top1cc by inducing SSBs or producing active intermediates that covalently bind to purines [62,63]. Camptothecin and other anticancer topoisomerase I inhibitors can trap Top1cc via specific binding to topoisomerase I-DNA complexes [64]. *DD1I* expression is increased in many cancer cell lines [65]. Thus, we predict that cancers may resist topoisomerase I inhibitors via *RAD23* and *DD11* upregulation.

*RAD52* encodes the key player in yeast DNA homologous recombination. Little is known about *RAD52* transcriptional regulation, since it is considered to be regulated mainly at the post-translational level [66]. Previous studies have shown that *RAD52* is induced during meiosis [67], upon exposure to DNA-damaging agents such as MMS, X-rays, and UV [68], and in proteasome mutants (*pup1-1*, cim5-1, and pre1-1 pre2-2) [69]. *RAD52* overexpression, mediated by the strong constitutive *ENO1* and controllable *GAL1* promoters, does not significantly alter yeast survival upon acute MMS exposure [70]. Our results corroborate these observations and show that an additional copy of the *RAD52* gene provided by a plasmid only slightly increases yeast resistance to chronic MMS stress (Figure 3h). However, an additional *RAD52* copy provides hyper-resistance to 4-NQO (Figure 3h). Our data suggest that *RAD52* overexpression plays an important role in 4-NQO resistance.

Yeast is a convenient model for mechanistic studies on cellular responses to DNA-damaging anticancer drugs [71]. Therefore, our results might have significance for therapeutic applications. There are obvious differences in the molecular mechanisms by which yeast and mammalian cells cope with DNA damage; however, despite these differences, yeast may still serve as a useful model for research into HDR-mediated cellular responses to anticancer drugs. This is exemplified by the similar mechanism by which curcumin acts on yeast and multiple myeloma (MM) cells. Curcumin is an HDAC inhibitor that, similar to DHC, represses yeast *RAD52* [72]. The bortezomib, melphalan and prednisone (VMP) regimen that includes melphalan, a DNA-alkylating and cross-linking drug, has been reported to be highly effective in the initial treatment of MM [73]. However, refractory or relapsed MM is still a problem. The mechanisms of MM resistance to melphalan may include the NFκB-dependent overexpression of DNA repair genes belonging to the Fanconi anaemia (FA) and BRCA pathways of HDR [74]. Curcumin, by inhibiting the NFκB [75] or FA/BRCA [76] pathways, reverses the multidrug-resistant phenotype of MM. As such, curcumin has been proposed as a component of combinatorial therapies for multidrug-resistant MM [77]. In the case of DHC, its synthetic lethality toward BRCA1-deficient breast cancer lines [78] also indicates its potential in combinatorial cancer therapy. Therefore, our *RAD52*-overexpressing proteasome mutants may serve as models for research into HDR inhibitors that reverse the multidrug-resistant phenotypes of hard-to-treat malignancies.

## 4. Materials and Methods

### 4.1. Yeast Strains

The wild-type yeast strain BY4742 and its mutant derivatives *rpn4*-Δ, *rad23*-Δ, *mag1*-Δ and *rad52*-Δ were obtained from Euroscarf (Oberursel, Germany). The mutant strains with the deregulated essential proteasomal subunits YPL and YRL were created earlier [19,24], and YRPL was created in this work. The genotypes of all used strains are described in Table S1.

The oligonucleotides and plasmids used in the work are described in Tables S2 and S3.

### 4.2. RT-PCR 

RT-PCR analysis was performed as described earlier [50]. Briefly, total RNA was isolated from yeast cell cultures grown to the logarithmic growth phase. cDNA was synthesized using RevertAid reverse transcriptase and oligo (dT) primers (Thermo Fisher Scientific, Waltham, MA, USA). RT-PCR was performed on a LightCycler 480-II instrument (Roche Diagnostics, Indianapolis, IN, USA) with Eva Green dye (Syntol, Moscow, Russia). Actin (*ACT1*) was used as a reference. The data were processed with LightCycler 480 Software, version 1.5, and Microsoft Excel (Redmond, WA, USA). The oligonucleotides are listed in Table S2.

### 4.3. β-. Galactosidase Assay

The lacZ reporter constructs consisted of the promoter of the tested gene translationally fused to the lacZ coding region. LacZ activity was measured as previously described [19]. Briefly, cells in the logarithmic growth phase were pelleted and lysed in Z-buffer (60 mM NaH_2_PO_4_, 40 mM Na_2_HPO_4_, 10 mM KCl, 1 mM MgSO_4_, 50 mM 2-mercaptoethanol) by vortexing with glass beads (Sigma, St. Louis, MO, USA). The cell lysates were clarified by centrifugation, mixed with ortho-nitrophenyl-β-galactoside (to 2 mg/mL) and incubated at 37 °C until a yellowish color formed. The reaction was stopped by the addition of 1 M Na_2_CO_3_. The total protein concentration and O-nitrophenol concentrations were measured spectrophotometrically using a NanoDrop ND-1000 (Thermo Fisher Scientific, Waltham, MA, USA).

### 4.4. DamID Assay

The activity of Dam methylase fused to Rpn4p was measured in a model system that we developed previously [34]. The activity of the Rpn4-tethered Dam methylase was measured by estimating the sensitivity of a nearby GATC site to MboI hydrolysis. The activity of the Dam–Rpn4 chimaera was normalized to the signal of Dam–Rpn4(C-A) lacking Rpn4-binding activity. Thus, the normalized signal reflects the specific binding of Rpn4.

### 4.5. Stress Resistance Test

Overnight cultures were diluted to OD600 = 1.0, and five-fold serial dilutions were then prepared. Subsequently, 2.5 or 5 μL of each dilution was spotted onto agar plates containing stressing agents, and the plates were then incubated at 30 °C for several days. The control plates were incubated without stressing agents.

### 4.6. Western Blot Analysis

Western blot analysis of yeast lysates was performed to assess the accumulation of polyubiquitinated proteins as described earlier [19]. Western blot analysis of the Rad52-3ha levels was performed in the same way. The pRad52-3ha plasmid-expressing Rad52, fused with the C-terminal 3xHA epitope from its native promoter, was assembled from PCR fragments by recombinational cloning [79]. PCR fragments used in the plasmid assembly were obtained using the primers listed in Table S2. The Rad52-3ha levels were determined using a primary mouse monoclonal anti-HA antibody (1:1000, Sigma, USA) and an anti-mouse secondary antibody (1:100,000, Abcam, Cambridge HQ, UK). Tubulin was used as a loading control and was detected using a primary rat monoclonal antibody (1:1000, Abcam, UK) and an anti-rat secondary antibody (1:100,000, Abcam, UK). The images obtained were analyzed using ImageJ (https://imagej.nih.gov/ij/). The protein intensities were normalized to the actin or tubulin intensity.

### 4.7. Proteasome Activity Measurement

20S proteasome activity was measured as described previously [24].

### 4.8. Mutation of Rpn4 Binding Sites Using the CRISPR/Cas9 System

Spacers were designed using CRISPOR (http://crispor.tefor.net/). Oligonucleotides encoding spacers (Table S2) were annealed and cloned into a BsaI-cut pCRCT vector [80]. Yeasts were co-transformed with pCRCT derivatives (Table S3) and PCR donor constructs. Each PCR donor carried an endonuclease (XhoI, XbaI or PstI) site instead of the GC-rich part of the PACE or PACE-like element. Yeast colonies were grown on synthetic selective media lacking uracil. Randomly picked colonies were screened for the presence of editing events using PCR followed by restriction analysis. The edited colonies were streaked to obtain single colonies, and the PACE mutations were verified by PCR followed by Sanger sequencing.

### 4.9. Gene Repression with the CRISPR/Cas9 System

Spacers targeting the SpyCas9 endonuclease to the PACE and PACE-like elements were designed using CRISPOR and cloned into pCRCT plasmids. The spacers were 14 nt long. At this length, a spacer allows SpyCas9 to bind but not to cut a DNA target [81]. Thus, SpyCas9 specifically bound to the PACE or PACE-like element should inhibit the Rpn4 interaction with the corresponding element.

## Supplementary Materials

Supplementary materials can be found at https://www.mdpi.com/1422-0067/21/21/8097/s1.

## Abbreviations

4-NQO	4-Nitroquinoline-1-oxide
AZE	Azetidine-2-carboxylic acid
MMS	Methyl methanesulfonate
PACE	Proteasome-associated control element
MACE	MAG1-associated control element
RACE	RPN8-associated control element
SSBs	DNA single-strand breaks
DSBs	DNA double-strand breaks
DDR	DNA damage response
BER	Base excision repair
NER	Nucleotide excision repair
MMR	Mismatch repair
HDR	Homology-directed repair
NHEJ	Non-homologous end joining
MMEJ	Microhomology-mediated end joining
SUMO	Small ubiquitin-like modifier
CRISPR	Clustered regularly interspaced short palindromic repeats
PAM	Protospacer-adjacent motif
Cas9	CRISPR-associated protein 9
sgRNA	Single guide RNA
RT-PCR	Real-time polymerase chain reaction
UV	Ultraviolet
DHC	Dihydrocoumarin
TopIcc	Topoisomerase I-DNA covalent complex
MM	Multiple myeloma
VMP	Bortezomib, melphalan, and prednisone regimen
HDAC	Histone deacetylase
FA	Fanconi anaemia
