# Supplementary material for: Yeast Rpn4 Links the Proteasome and DNA Repair via RAD52 Regulation
Source: Int J Mol Sci. 2020 Oct 30;21(21):8097. doi: 10.3390/ijms21218097 (PMC7672625; doi:10.3390/ijms21218097)
Supplement: Supplementary file 1 [file ijms-21-08097-s001.zip › Supplementary_captions_v2.docx]

Supplementary Figure 1. Developed western blot image quantified in Figure 1(b).

Supplementary Figure 2. RT-PCR was used to determine the mRNA expression levels of *DEF1*, *MSH3*, and *SSL2* in the wild-type and *rpn4-Δ* strains under normal conditions or after 4-NQO treatment at a final concentration of 2 µg/ml for 45 min.

Supplementary Figure 3. 20S proteasome activity measured in the cell lysates of *RAD52* mutant strains.

Supplementary Figure 4. The fully developed western blot image presented in Figure 4(b).

Supplementary Figure 5. The presence of mutations of Rpn4 binding sites in the promoters of PRE1 or RAD52 verified by PCR amplification of the mutated promoters followed by restriction analysis.

Supplementary Figure 6. The mRNA expression levels of PRE1 and RAD52 in mutant strains with mutated Rpn4 binding sites.

Supplementary Table 1. Strains used in the study.

Supplementary Table 2. Oligonucleotides used in the study.

Supplementary Table 3. Plasmids used in the study.

Supplementary Table 4. Potential Rpn4 target genes that are differentially expressed upon DNA damage stress.
