# Supplementary material for: Yeast Rpn4 Links the Proteasome and DNA Repair via RAD52 Regulation
Source: Int J Mol Sci. 2020 Oct 30;21(21):8097. doi: 10.3390/ijms21218097 (PMC7672625; doi:10.3390/ijms21218097)
Supplement: Supplementary file 1 [file ijms-21-08097-s001.zip › Supplementary_Tables_1_3-v2.docx]

**Table S1. Strains used in the study.**

| **Cloning of CRISPR plasmids** | | |
| --- | --- | --- |
| **BY4742** | MAT α; his3D1; leu2D0; lys2D0; ura3D0 | Euroscarf (Germany) |
| ***rpn4*-Δ** | BY4742 YDL020c::kanMX4 | Euroscarf (Germany) |
| ***mag1*-Δ** | BY4742 YER142C::kanMX4 | Euroscarf (Germany) |
| ***rad23*-Δ** | BY4742 YEL037C::kanMX4 | Euroscarf (Germany) |
| ***rad52*-Δ** | BY4742 YML032c::kanMX4 | Euroscarf (Germany) |
| **YPL** | BY4742 *pre1-8,* nat+ | [1] |
| **YRL** | BY4742 *rpt5–76* | [2] |
| **YRPL** | BY4742 *pre1-8*, *rpt5–76* | This study |
| **MAG1-pM** | BY4741 *mag1-7* | This study |
| **MAG1-pdM** | BY4741 *mag1-78* | This study |
| **mRAD23** | BY4741 *rad23-9* | This study |
| **mRAD52** | BY4741 *rad52-30* | This study |
| **RMdM** | BY4741 *mag1-7, rad23-9* | This study |
| **YPL, RMdM** | BY4741 *pre1-8*, *mag1-7, rad23-9* | This study |
| **YPL, mRAD52** | BY4742 *pre1-8,* nat+, *rad52-30* | This study |

**Table S2. Oligonucleotides used in the study.**

| **Cloning of CRISPR plasmids** | | | |
| --- | --- | --- | --- |
| **pCRCT-PRE1-P-gRNA-F** | | CCAAAACGTCGCAGCGAAATCTTTACGGGTTTT | PACE mutation in the *PRE1* gene promoter |
| **pCRCT-PRE1-P-gRNA-R** | | CTCTAAAACCCGTAAAGATTTCGCTGCGACGTT |  |
| **pCRCT-RPT5-P-gRNA-F** | | CCAAAACGTAGTTACCCGGCCAATATGGTTTT | PACE mutation in the *RPT5* gene promoter |
| **pCRCT-RPT5-P-gRNA-R** | | CTCTAAAACCATATTGGCCGGGTAACTACGTT |  |
| **pCRCT-RAD52-R-gRNA-F** | | CCAAAACTTGCATTTGTTGATTCTCAGGTTTT | RACE mutation in the *RAD52* gene promoter |
| **pCRCT-RAD52-R-gRNA-R** | | CTCTAAAACCTGAGAATCAACAAATGCAAGTT |  |
| **pCRCT-RAD23-M-gRNA-F** | | CCAAAACAATCACTATACACGGCTCGGGTTTT | MACE mutation in the *RAD23* gene promoter |
| **pCRCT-RAD23-M-gRNA-R** | | CTCTAAAACCCGAGCCGTGTATAGTGATTGTT |  |
| **pCRCT-MAG1-pM-gRNA-F** | | CCAAAACGATGAATTTACAGGGCGGGGGTTTT | Proximal MACE mutation in the *MAG1*-*DDI1* promoter |
| **pCRCT-MAG1-pM-gRNA-R** | | CTCTAAAACCCCCGCCCTGTAAATTCATCGTT |  |
| **pCRCT-PRE1-Repr-1-F** | | CCAAAACGCGAAATCTTTACGGGTTTT | Repression of *PRE1* gene via PACE binding |
| **pCRCT-PRE1-Repr-1-R** | | CTCTAAAACCCGTAAAGATTTCGCGTT |  |
| **pCRCT-RPT3-Repr-F** | | CCAAAACGCCACCTTACTTTCGTT TT | Repression of *RPT3* gene via PACE binding |
| **pCRCT-RPT3-Repr-R** | | CTCTAAAACGAAAGTAAGGTGGCGTT |  |
| **pCRCT-RAD52-Repr-F** | | CCAAAACTTGTTGATTCTCAGGTTTT | Repression of *RAD52* gene via RACE binding |
| **pCRCT-RAD52-Repr-R** | | CTCTAAAACCTGAGAATCAACAAGTT |  |
| **Creation of DNA repair templates for *S. cerevisiae* genome editing** | | | |
| MAG1-pMACE-XbaI-F | | ATGTAAGAATTCCCGTTCTAGACCCGCCCTGTAAAT | Mutation of proximal (pMACE) or distal (dMACE) MACE sites in the *MAG1-DDI1* bidirectional promoter |
| MAG1-pMACE-XbaI-R | | ATTTACAGGGCGGGTCTAGAACGGGAATTCTTACAT |  |
| MAG1-dMACE-XhoI-F | | GCCCTGTAAATTCATCTCGAGCGAAAAGATAATAATACCA |  |
| MAG1-dMACE-XhoI-R | | TGGTATTATTATCTTTTCGCTCGAGATGAATTTACAGGGC |  |
| MAG1-template-F | | CGAGTGTATTAGGGACTTGAG |  |
| MAG1-template-R | | GTAATATAAGTCGTGTTTTGTTTTGTC |  |
| RAD23-MACE-XbaI-F | | CGGCTCTCTAGAGAAATTGAAATTTTTTTATTTTC | Mutation of the MACE site in the *RAD23* promoter |
| RAD23-MACE-XbaI-R | | CAATTTCTCTAGAGAGCCGTGTATAGTGATTGAT |  |
| RAD23-template-F | | AGAGAGGATCCGCTCGGTTTTTTAGTG |  |
| RAD23-template-R | | AGAGAGGATCCCCCATCTTTTAGCCCGCATTC |  |
| PRE1-PACE-PstI-F | | GAAATCTTTACCTGCAGAAATAAAGAAAAGTGAATATTGAACA | Mutation of the PACE site in the *PRE1* promoter |
| PRE1-PACE-PstI-R | | TTTCTTTATTTCTGCAGGTAAAGATTTCGCTGCGAAAG |  |
| PRE1-template-F | | TGTGGTAGTAAATTAAATAGGC |  |
| PRE1-template-R | | TTGTGACTGCCTTAGAAGAC |  |
| RPT5-PACE-XbaI-F | | GTAGTTACCCGGCCAATATGTTCTAGAAAAAATGAA | Mutation of the PACE site in the *RPT5* promoter |
| RPT5-PACE-XbaI-R | | TAAAATATACATTAATTCATTTTTTCTAGAACATATTGG |  |
| RPT5-template-F | | GAAGAAAGAAGCTTCCAACCAGTGATGAG |  |
| RPT5-template-R | | GAAGAAAGGGATCCTTCCTTATTGTCCTTAATCTTC |  |
| **Oligonucleotides used in the lacZ and DamID experiments** | | | |
| **-258RAD52HindIII-F** | GAGAGAAGCTTCTGCTTGCCCTGTAATG | | Cloning lacZ-RAD52 reporter constructs, *RAD52* locus cloning |
| **+195RAD52BamHI-R** | AGAGAGGATCCGCCATTATATCCAAAG | |  |
| **RAD52dRACE-F** | CGTTTTTAAGCTATTGAGAATCAAC | |  |
| **RAD52dRACE-R** | GTTGATTCTCAATAGCTTAAAAACG | |  |
| **RAD52-RACE-XbaI-F** | TTAAGCTATTTTTCTAGAGAGAATCAACAAATGCAAAC | |  |
| **RAD52-RACE-XbaI-R** | TTGTTGATTCTCTCTAGAAAAATAGCTTAAAAACGCCAT | |  |
| **RAD52 +577-F** | CATCCTTTTATCATTCGTAC | | DamID |
| **RAD52 +577-R** | CAGTTGAACGACAATTTGTC | |  |
| **ADH1-DamID-F** | CAAGAACCGTTCAACCATTTG | |  |
| **ADH1-DamID-R** | CGTTAAATACTCTGGTGTCTG | |  |
| **Measurement of gene expression by RT-PCR** | | | |
| **RAD52-ex-RT-F** | TAGGCACACCGTTGATCAGAC | | RT-PCR *RAD52* |
| **RAD52-ex-RT-R** | CCCAGTTCTTTATCATTGTTCT | |  |
| **MAG1-ex-RT-F** | CCTTTCAGATAAGCCAGAATT | | RT-PCR *MAG1* |
| **MAG1-ex-RT-R** | TTTTCCATTATGTCGTCGTC | |  |
| **RAD23-ex-RT-F** | GTGCTAGATATCCTCAATTACG | | RT-PCR *RAD23* |
| **RAD23-ex-RT-R** | TCCTCTCCTTCCACCATATCAT | |  |
| **DDI-ex-RT-F** | GGCTTGTGTGGACTTAAAGGAA | | RT-PCR *DDI1* |
| **DDI-ex-RT-R** | TGTAACTGACGTTGGTGCTGGA | |  |
| **PRE1-ex-RT-F** | ACTACTGAGGAGGGTTTAG | | RT-PCR *PRE1* |
| **PRE1-ex-RT-R** | CTTATGCCATCTTTATCCACGA | |  |
| **SSL2-ex-RT-F** | GGTGATGACGCTGACAATTC | | RT-PCR *SSL2* |
| **SSL2-ex-RT-R** | GCAAGACCAGACAATGAACC | |  |
| **DEF1-ex-RT-F** | ACCTGTTAACCCACAACAAC | | RT-PCR *DEF1* |
| **DEF1-ex-RT-R** | TAGCCATATGGTACACCTTG | |  |
| **MSH3-ex-RT-F** | CATCCCATAACTATTATAAGTC | | RT-PCR *MSH3* |
| **MSH3-ex-RT-R** | CTCCGTTAATAAGGAATTATC | |  |
|  | **Assembly of the pRAD52-3ha plasmid** | |  |
| **pRS-pRAD52-rec-F** | GTACCGGGCCCCCCCTCGAGGTCGACGGTATCGATAAGCTTATGATCAAAAGGGAAACTAGG | |  |
| **RAD52-3HA-rec-F** | CTGCATGCACGCAAGCCTACTGCAAAGGCTAATATTGGTTATG | |  |
| **3HA-RAD52-rec-R** | CATAACCAATATTAGCCTTTGCAGTAGGCTTGCGTGCATGCAG | |  |
| **pRS-tCYC-rec-R** | CTCCACCGCGGTGGCGGCCGCTCTAGAACTAGTGGATCCAGCTTGCAAATTAAAGCCTTC | |  |

**Table S3. Plasmids used in the study.**

|  | |
| --- | --- |
| **YcpLac36**  **pRS426**  **pRAD52-3ha** | Low-copy episomal yeast expression plasmid [1]  Medium-copy episomal yeast expression plasmid [3]  pRS426 derivative encoding Rad52 fused with C-terminal 3xHA epitope under the control of the native *RAD52* promoter |
| **YcpLac50-RPT6** | Low-copy episomal yeast expression plasmid with a reporter *RPT6-lacZ* translational fusion under the control of the native *RPT6* promoter [4] |
| **pCRCT** | Low-copy episomal yeast expression plasmid expressing component of the CRISPR/Cas9 system: SpCas9 and tracrRNA and crRNA |
| **pCRCT-PRE1** | pCRCT derivative expressing 20 nt sgRNA against PACE in the *PRE1* promoter |
| **pCRCT-RPT5** | pCRCT derivative expressing 20 nt sgRNA against PACE in the *RPT5-RPO31* bidirectional promoter |
| **pCRCT-RAD52** | pCRCT derivative expressing 20 nt sgRNA against RACE in the *RAD52* promoter |
| **pCRCT-RAD23** | pCRCT derivative expressing 20 nt sgRNA against MACE in the *RAD23* promoter |
| **pCRCT-MAG1** | pCRCT derivative expressing 20 nt sgRNA against proximal MACE in the *MAG1-DDI1* bidirectional promoter |
| **pCRCT-rPRE1** | pCRCT derivative expressing 14 nt sgRNA against PACE in the *PRE1* promoter |
| **pCRCT-rRPT3** | pCRCT derivative expressing 14 nt sgRNA against PACE in the *RPT3* promoter |
| **pCRCT-rRAD52** | pCRCT derivative expressing 14 nt sgRNA against RACE in the *RAD52* promoter |
| **pCRCT-rRAD23** | pCRCT derivative expressing 14 nt sgRNA against MACE in the *RAD23* promoter |
| **pCRCT-rMAG1** | pCRCT derivative expressing 14 nt sgRNA against proximal MACE in the *MAG1-DDI1* bidirectional promoter |
| **pRAD52** | YcpLac36 derivative bearing the *RAD52* gene under the control of the native promoter |
| **pRAD52-LacZ** | YcpLac50-RPT6 derivative encoding reporter *RAD52*-*lacZ* translational fusion under the control of the native *RAD52* promoter |
| **pRmut** | YcpLac50-RPT6 derivative encoding reporter *RAD52*-*lacZ* translational fusion under the control of the mutant *RAD52* promoter with RACE to XbaI mutation |
| **pRΔ** | YcpLac50-RPT6 derivative encoding reporter *RAD52*-*lacZ* translational fusion under the control of the mutant *RAD52* promoter with RACE deletion |
| **pRPN4ΔNN** | YcpLac36 derivative encoding stabilized Rpn4 with deletion of the N-terminal degradation signal and the N-terminal acidic domain (NAD). Mutant *RPN4* gene is under the control of the native *RPN4* promoter [1]. |
| **pRPN4ΔN6R** | YcpLac36 derivative encoding stabilized Rpn4 with deletion of the N-terminal degradation signal and mutation of six lysines to arginines in the N-terminal region. The mutant *RPN4* gene is under the control of the native *RPN4* promoter [1]. |

**Supplementary references**

1. Karpov, D. S.; Spasskaya, D. S.; Tutyaeva, V. V.; Mironov, A. S.; Karpov, V. L. Proteasome inhibition enhances resistance to DNA damage via upregulation of Rpn4-dependent DNA repair genes. *FEBS letters* **2013,** 587, (18), 3108-14.

2. Karpov, D. S.; Spasskaya, D. S.; Nadolinskaia, N. I.; Tutyaeva, V. V.; Lysov, Y. P.; Karpov, V. L. Deregulation of the 19S proteasome complex increases yeast resistance to 4-NQO and oxidative stress via upregulation of Rpn4- and proteasome-dependent stress responsive genes. *FEMS yeast research* **2019,** 19, (2).

3. Christianson, T.W.; Sikorski, R.S.; Dante, M.; Shero, J.H.; Hieter, P. Multifunctional yeast high-copy-number shuttle vectors. *Gene* **1992**, 110, (1), 119-122.

4. Osipov, S.; Tutyaeva, V.; Preobrazhenskaya, O.; Karpov, V. A rapid method for liquid β-galactosidase reporter assay in Saccharomyces cerevisiae. *World Journal of Microbiology and Biotechnology* **2011,** 27, (5), 1255-1259.
