# Supplementary figures and images for: Yeast Rpn4 Links the Proteasome and DNA Repair via RAD52 Regulation
Source: Int J Mol Sci. 2020 Oct 30;21(21):8097. doi: 10.3390/ijms21218097 (PMC7672625; doi:10.3390/ijms21218097)

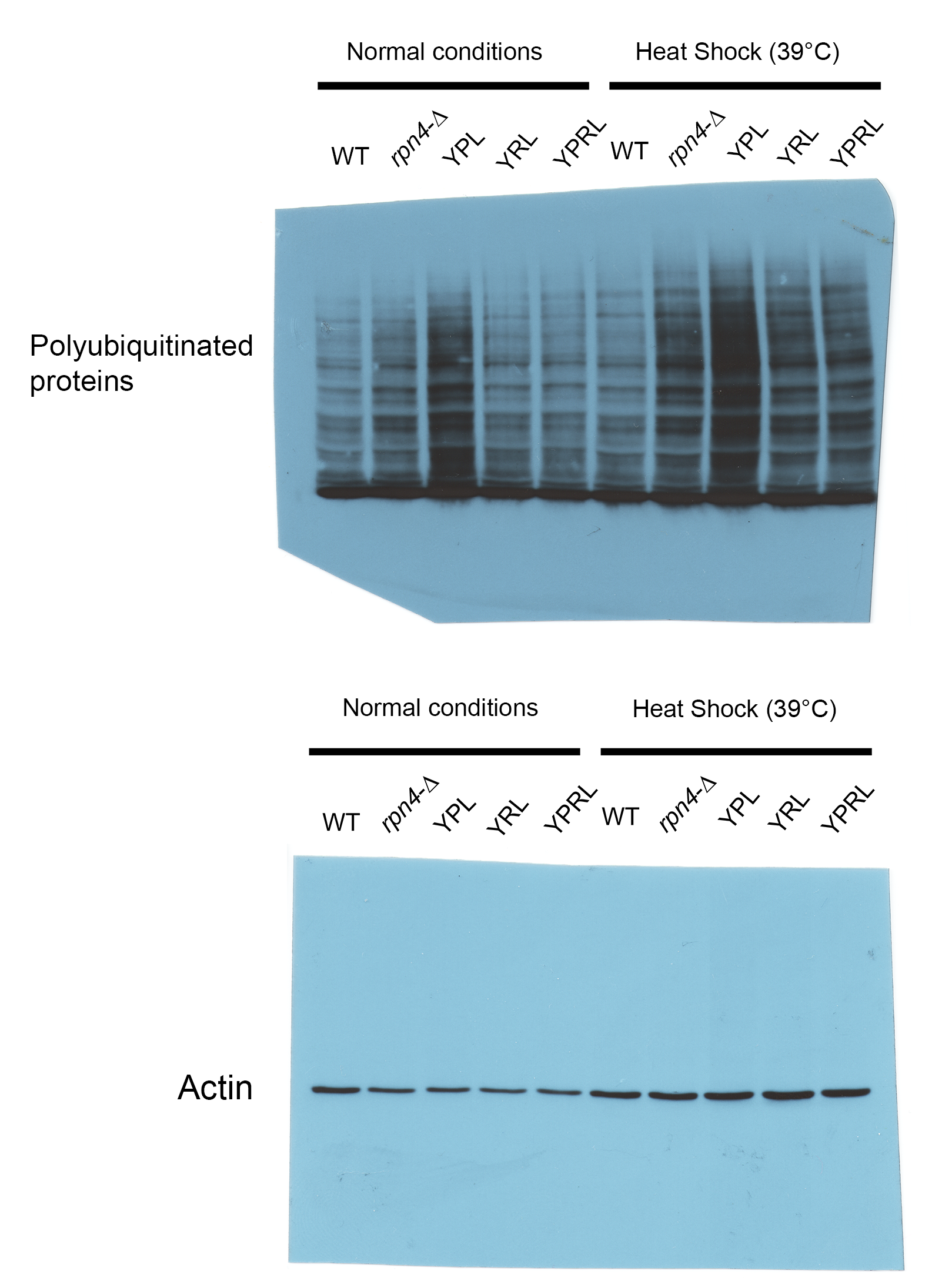

Supplement: Supplementary file 1 [file ijms-21-08097-s001.zip › Supplementary figure 1_.tif]

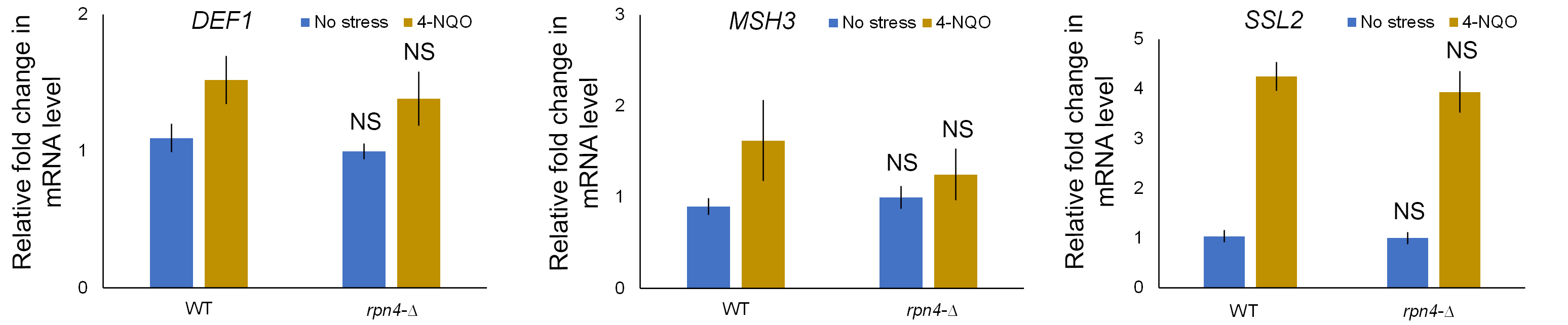

Supplement: Supplementary file 1 [file ijms-21-08097-s001.zip › Supplementary Figure 2_.tif]

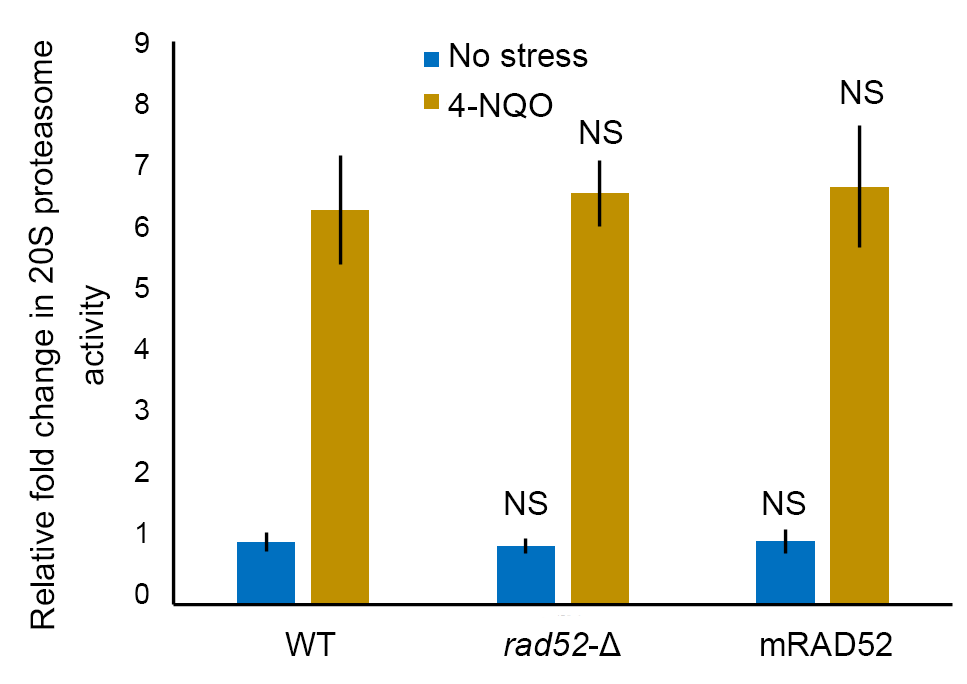

Supplement: Supplementary file 1 [file ijms-21-08097-s001.zip › Supplementary Figure 3_.tif]

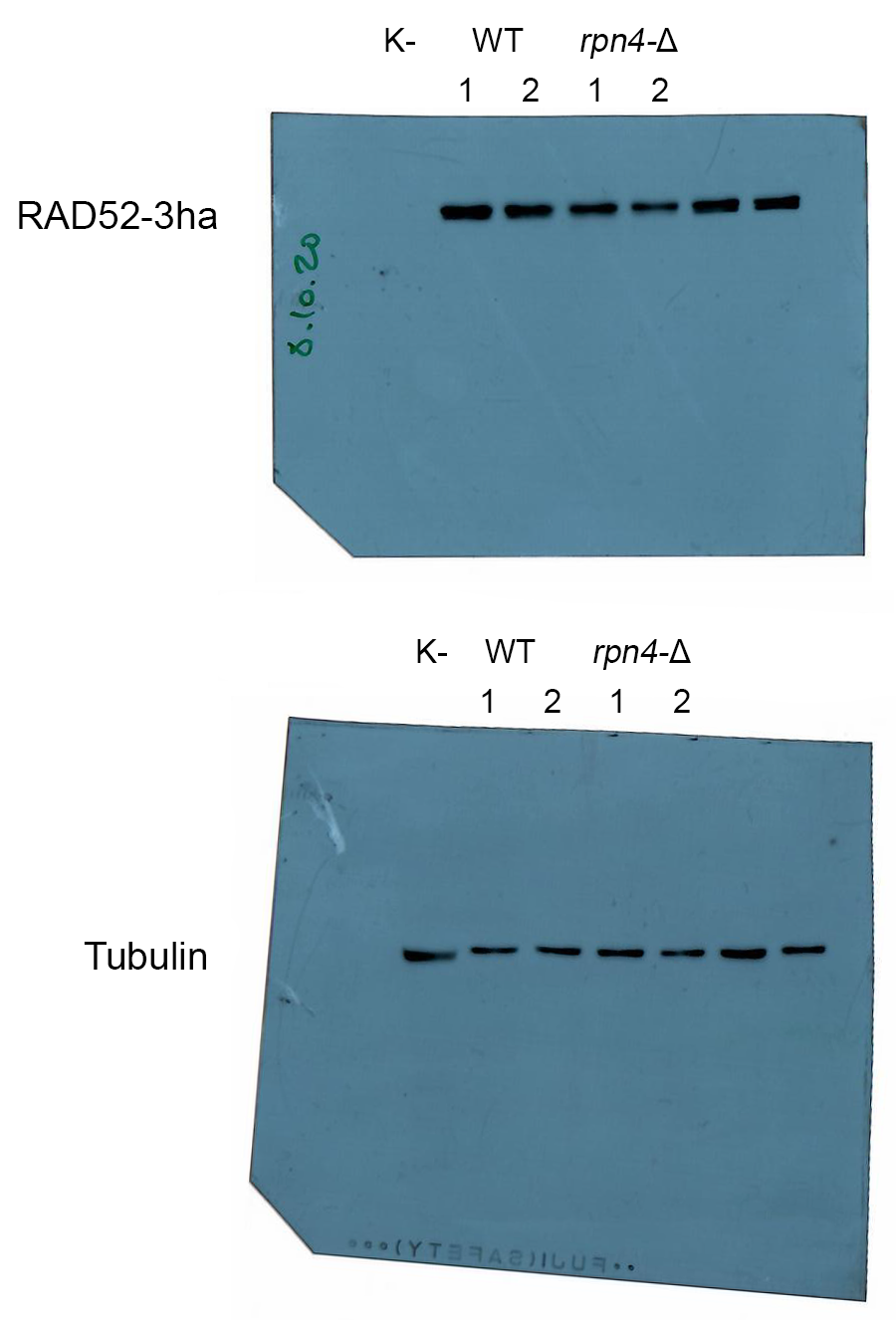

Supplement: Supplementary file 1 [file ijms-21-08097-s001.zip › Supplementary Figure 4_.tif]

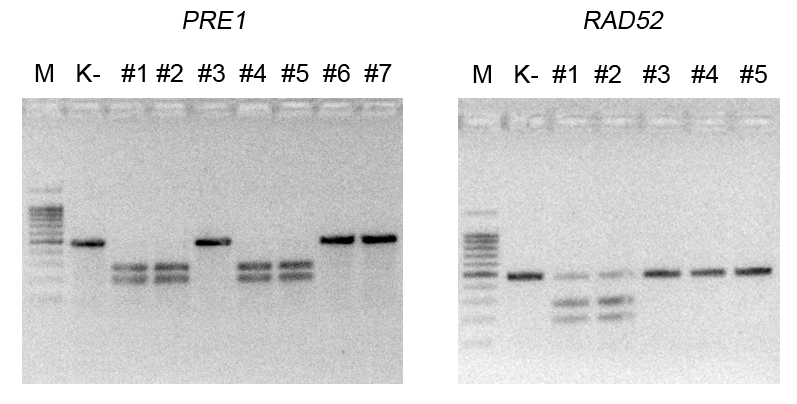

Supplement: Supplementary file 1 [file ijms-21-08097-s001.zip › Supplementary figure 5_.tif]

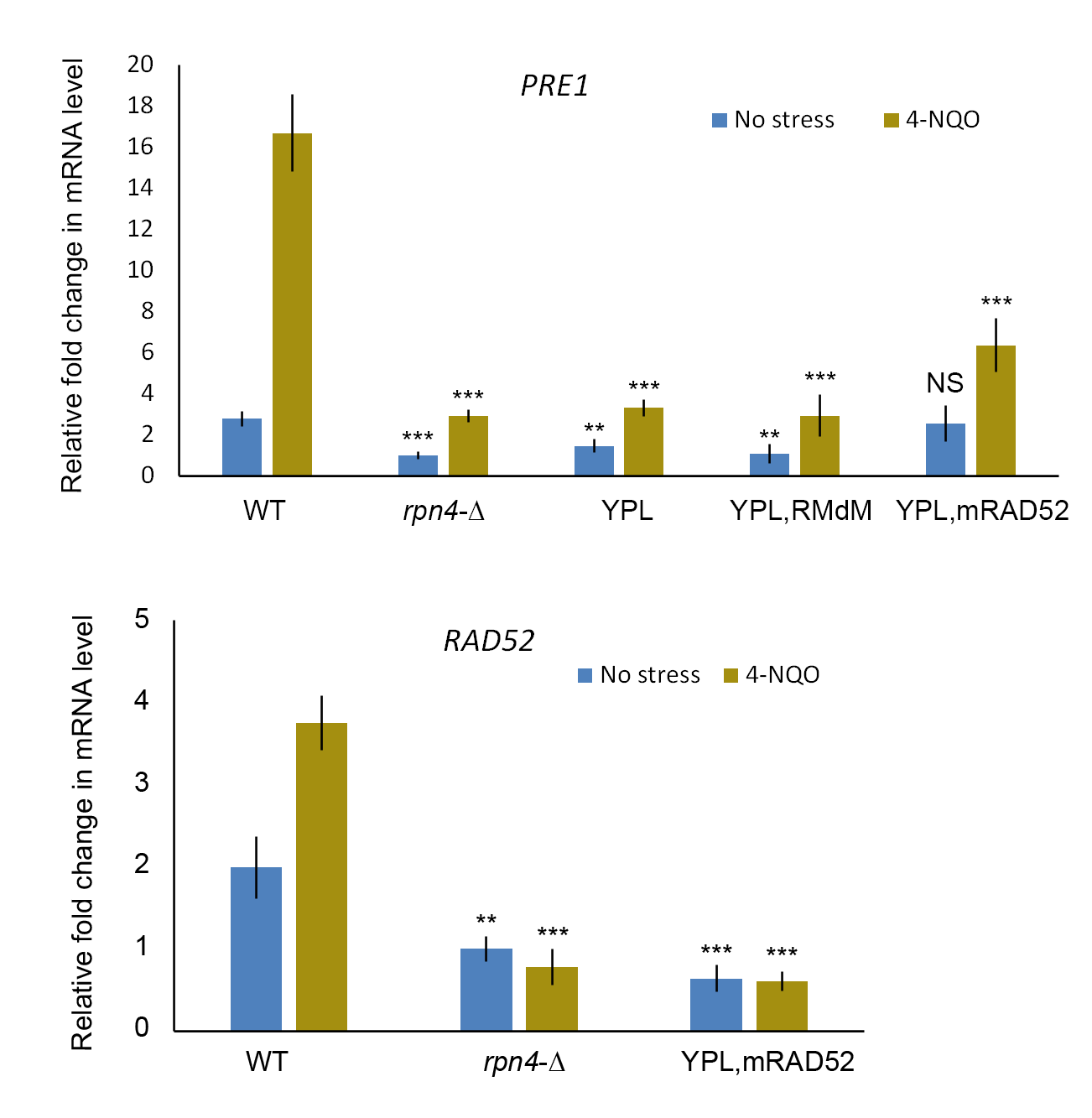

Supplement: Supplementary file 1 [file ijms-21-08097-s001.zip › Supplementary Figure 6_.tif]
